# Small stepping motion of processive dynein revealed by load-free high-speed single-particle tracking

**DOI:** 10.1038/s41598-020-58070-y

**Published:** 2020-01-23

**Authors:** Jun Ando, Tomohiro Shima, Riko Kanazawa, Rieko Shimo-Kon, Akihiko Nakamura, Mayuko Yamamoto, Takahide Kon, Ryota Iino

**Affiliations:** 1 Institute for Molecular Science, National Institutes of Natural Sciences, Okazaki, 444-8787 Aichi Japan; 2 The Graduate University for Advanced Studies (SOKENDAI), Hayama, 240-0193 Kanagawa Japan; 3 Department of Biological Sciences, Graduate School of Science, The University of Tokyo, Hongo, 113-0033 Tokyo Japan; 4 Department of Biological Sciences, Graduate School of Science, Osaka University, Toyonaka, Osaka, 560-0043 Japan

**Keywords:** Biophysics, Single-molecule biophysics

## Abstract

Cytoplasmic dynein is a dimeric motor protein which processively moves along microtubule. Its motor domain (head) hydrolyzes ATP and induces conformational changes of linker, stalk, and microtubule binding domain (MTBD) to trigger stepping motion. Here we applied scattering imaging of gold nanoparticle (AuNP) to visualize load-free stepping motion of processive dynein. We observed artificially-dimerized chimeric dynein, which has the head, linker, and stalk from *Dictyostelium discoideum* cytoplasmic dynein and the MTBD from human axonemal dynein, whose structure has been well-studied by cryo-electron microscopy. One head of a dimer was labeled with 30 nm AuNP, and stepping motions were observed with 100 μs time resolution and sub-nanometer localization precision at physiologically-relevant 1 mM ATP. We found 8 nm forward and backward steps and 5 nm side steps, consistent with on- and off-axes pitches of binding cleft between αβ-tubulin dimers on the microtubule. Probability of the forward step was 1.8 times higher than that of the backward step, and similar to those of the side steps. One-head bound states were not clearly observed, and the steps were limited by a single rate constant. Our results indicate dynein mainly moves with biased small stepping motion in which only backward steps are slightly suppressed.

## Introduction

Cytoplasmic dynein is a dimeric motor protein which processively moves along microtubules in a cell^1^. Dynein transports cargo toward the minus end of the microtubule^2,3^, opposite direction to the conventional kinesin motor protein^4^, and plays important roles on various cellular activities such as axonal transport^2,3^ and cell division^5^. Dynein has a large and complex structure, consisting of ring-shaped motor domain (head), linker, tail, stalk, and microtubule binding domain (MTBD)^1,6,7^. The head has six AAA^+^ (ATPase associated with various cellular activities) modules, AAA1 to AAA6^6^. Among them, AAA1 to AAA4 bind ATP, and AAA1, AAA3, and AAA4 hydrolyze ATP. AAA1 is the main ATP hydrolysis site which drives dynein motion. ATP hydrolysis at AAA3 (and perhaps AAA4) is considered to have a regulatory role. The tail dimerizes two dynein molecules, and also binds with the other subunits and cargo. The linker connects the head and tail, and changes its conformation to provide power stroke that mainly drives dynein motion. The stalk extends from the head, and has a 14-nm long coiled-coil structure. The MTBD exists at the tip of the stalk, and binds to the cleft between αβ-tubulin dimers on the surface of microtubule^8,9^. Complex communications among these domains drive the stepping motion of dynein^1,6,7^.

To understand the stepping mechanism of dynein, not only the structural analysis at the atomic level, but also single-molecule analysis of dynamics is necessary. Previously, single-molecule analysis of processive dynein has been conducted extensively with fluorescence imaging^10^ or optical tweezers^11,12^. In these studies, stepping motion of a single dynein molecule labeled with fluorescent dye or polystyrene bead has been tracked. Furthermore, recently, labeling with quantum dot (QD) or fluorescent dye has been successfully applied for simultaneous dual-color imaging of two heads of a single dynein molecule^13,14^. These landmark studies have unveiled unique properties of dynein motility. One significant feature is that in addition to the forward steps along the microtubule long axis (on-axis), dynein often shows the backward steps and also side steps along the microtubule short axis (off-axis)^10^. Furthermore, simultaneous dual-color imaging indicated that stepping motion of each head is uncoordinated^13,14^. These features are largely different from well-studied dimeric motor protein kinesin-1, which moves along a single protofilament of the microtubule with highly-coordinated hand-over-hand manner without backward and side steps^15^. Recently, structural basis determining the stepping direction of dynein has been also reported with engineered molecules^16^.

Although the stepping mechanism of dynein has been substantially revealed, high-speed imaging of load-free fast motion of the single head in a dimer will be still very helpful to understand the elementary stepping behaviors in detail. In addition, it will be important to observe fast motion of dynein at a physiologically-relevant high ATP concentration ([ATP]), because multiple AAA modules of the head may regulate the stepping behavior depending on [ATP]. To achieve high-speed single-molecule imaging, fluorescent dye and even QD are not suitable as probes, because photobleaching limits numbers of photons obtained and prevents further improvement of the time resolution. On the other hand, polystyrene bead does not suffer from photobleaching, and single-molecule imaging and manipulation with microsecond time resolution is possible. However, sub-micron size of the polystyrene bead is too large to track load-free fast motions.

In this study, by using scattering imaging of gold nanoparticle (AuNP), we visualized load-free fast stepping motion of artificially-dimerized chimeric dynein with 100 µs time resolution and sub-nanometer localization precision at physiologically-relevant 1 mM ATP. Since AuNP strongly scatters light at its plasmon resonance wavelength without suffering from photobleaching and blinking, it has been used as a probe of single-molecule imaging of linear and rotary motor proteins *in vitro*^17–25^ and imaging of intracellular cargo transport^26–28^. We analyzed trajectory of the stepping motion of the chimeric dynein in detail, including the step size, preference of the step direction, and dwell time, and revealed that this chimeric dynein moves with biased small stepping motion in which only backward steps are slightly suppressed compared to forward and side steps.

## Results and Discussion

### Chimeric dynein construct and single-molecule imaging of stepping motion

**Figure 1:**
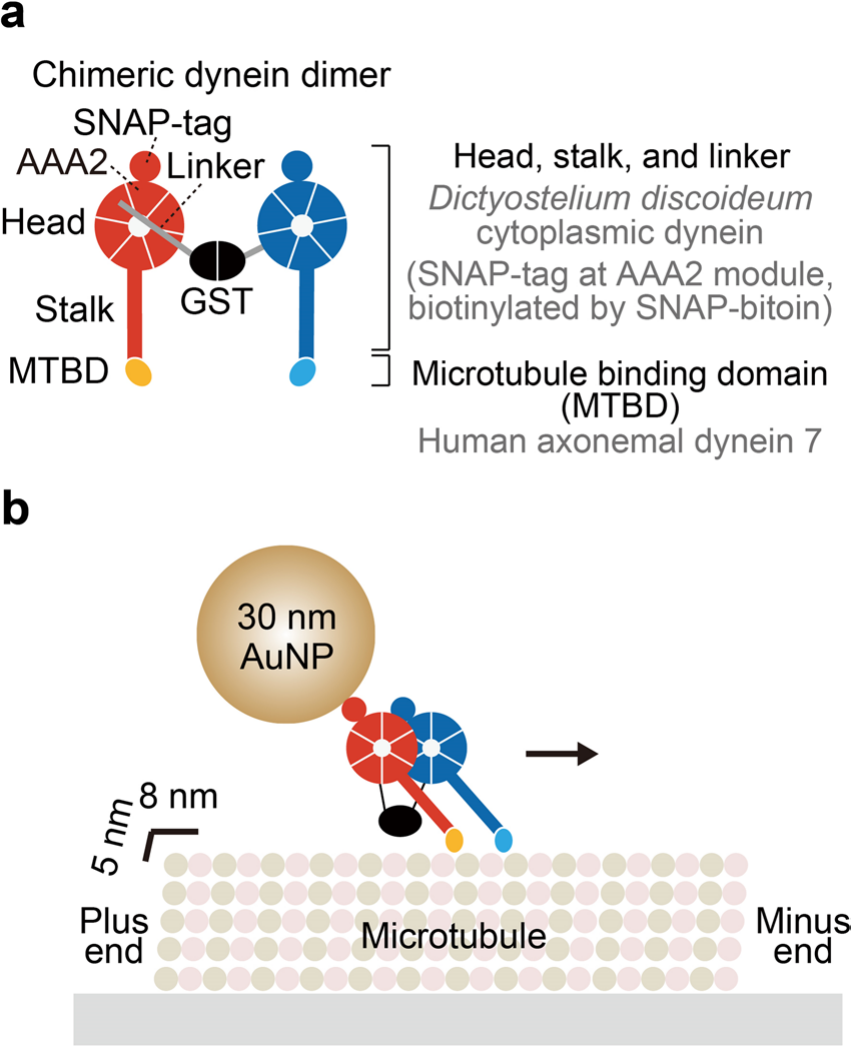
Artificially-dimerized chimeric dynein and experimental system used in this study. (**a**) Schematic depiction of domain architecture of the chimeric dynein. The motor domain (head), linker, and stalk are from *D. discoideum* dynein. The MTBD is from human axonemal dynein heavy chain 7 to enhance affinity to the microtubule. The tail was replaced with GST to form stable dimer. SNAP-tag was introduced into AAA2 module of the head and biotinylated (labeling ratio was 0.4 per head). (**b**) Schematic depiction of experimental system. The microtubules were immobilized on the glutaraldehyde-modified glass surface. 30 nm AuNP-labeled dynein was then introduced, and stepping motion was observed in the presence of ATP.

In this study, we used a chimeric dynein, whose structure on microtubule has been well-studied by cryo-electron microscopy^29^. It has the head, linker, and stalk from *Dictyostelium discoideum* cytoplasmic dynein and the MTBD from human axonemal dynein, and glutathione S-transferase (GST) tag replaced with the tail to form a stable dimer (Fig. 1a)^29^. This chimeric construct also has SNAP-tag introduced into the AAA2 module of the head for AuNP labeling. Because of the replacement of the MTBD, chimeric dynein showed higher affinity to the microtubule compared with native dynein and facilitated cryo-electron microscopic structural study^29^. Also note that it has been reported previously that both native and chimeric dyneins with SNAP-tag showed processive motion^29,30^. Velocities of native and chimeric dyneins, labeled with fluorophore, were 610 ± 20 nm/s and 190 ± 20 nm/s (mean ± standard error), respectively^29^. Lower velocity of the chimeric dynein than the native one was attributed to the high affinity of the MTBD to the microtubule. Hereafter, we refer this artificially-dimerized chimeric dynein with SNAP-tag as dynein for simplicity, unless otherwise noted. The SNAP-tag was biotinylated with SNAP-biotin (labeling ratio was 0.4 per head), and then bound with streptavidin-coated 30 nm AuNP to visualize stepping motion (Fig. 1b).

**Figure 2:**
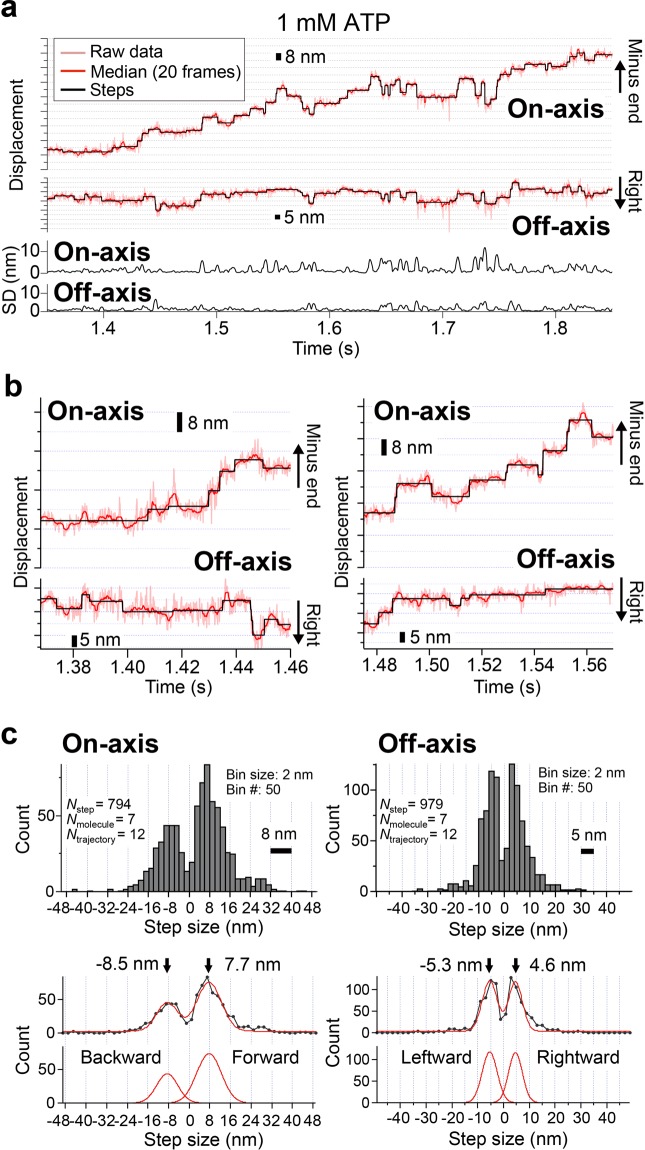
Trajectory and step size at 100 μs time resolution at 1 mM ATP. (**a**) Light red lines represent typical raw trajectories of centroid position of AuNP-labeled dynein along the microtubule long axis (on-axis) and short axis (off-axis). Red lines represent median-filtered trajectories (window size of 20 frames). Lower panel shows SD of the median-filtered trajectory along the on- and off-axes at each time frame t, calculated for t ± 20 frames. Black lines show steps and pauses in the median-filtered trajectories identified by the algorithm developed by Kerssemakers *et al*.^32^. (**b**) Enlarged views of the trajectory. (**c**) Distribution of step size along on- and off-axes. The peak positions of the distributions, determined by fitting with a sum of two Gaussians (red lines), were 7.7 nm, −8.5 nm, 4.6 nm, and −5.3 nm for forward, backward, rightward, and leftward directions, respectively.

Then, we conducted single-molecule imaging of stepping motion at 100 μs time resolution at 1 mM ATP, a physiologically-relevant [ATP]. For imaging of AuNP-labeled dynein, we used annular illumination total internal reflection dark-field microscopy^31^. In our experimental condition, 30 nm AuNP fixed on the glass surface showed localization precision of 0.7 nm at 100 μs time resolution. Figures 2a,b, and S1 show the typical trajectories of the centroid position of 30 nm AuNP attached to the dynein, along on- and off-axes of the microtubule. As previously reported with dynein labeled with fluorescent dye or QD^10,13,14,29^, AuNP-labeled dynein molecules showed processive motions with forward and backward steps in on-axis and side steps in off-axis. The motion in on-axis was clearly biased to the forward direction.

The velocity of AuNP-labeled dynein at 1 mM ATP was 117 ± 19 nm/s (mean ± standard error), which was lower than that of fluorophore-labeled same dynein construct in the previous study (190 ± 20 nm/s)^29^. This velocity difference would come from difference in the microtubule preparation with which GTP (present study) or GMPCPP (previous study)^29^ was used for polymerization. It has been reported that velocity of *D. discoideum* dynein on GTP-polymerized microtubule^30^ is lower than that on GMPCPP-polymerized microtubule^29^. In addition to the difference in the microtubule preparation, AuNP labeling may also slightly decrease the velocity of dynein.

### Step size and preference of step direction

Next, steps in the median-filtered trajectories (Fig. 2a, red lines, window size of 20 frames) along on- and off-axes were identified with the algorithm developed by Kerssemakers *et al*. (Fig. 2a, black lines on trajectories)^32^. The lower panel of Fig. 2a shows standard deviation (SD) of the median-filtered trajectory at each time frame t, calculated for t ± 20 frames. For both on- and off-axes, SD was found to be almost always few nanometers, indicating sufficient localization precision to resolve steps. Figure 2b shows enlarged views of the trajectory. The distribution of the step size along on-axis showed peaks at 7.7 nm for forward direction and −8.5 nm for backward direction (Fig. 2c, left). The distribution of the step size along off-axis showed peaks at 4.6 nm for rightward direction and −5.3 nm for leftward direction (Fig. 2c, right). These step sizes matched well with the pitches of the binding cleft between αβ-tubulin dimers on the microtubule surface (8 nm along on-axis and 5 nm along off-axis)^33–35^. The ratio of forward step to backward step along on-axis was 2.0, and the stepping direction was twice biased to the forward (Fig. 2c, left). On the other hand, steps along off-axis were not biased, with the ratio of 1.1 (leftward step to rightward step) (Fig. 2c, right).

In the previous single-molecule imaging, step size of head-labeled yeast dynein was highly variable between 8−32 nm along on-axis (for both forward and backward directions)^10,13,14,29^. Distribution of step size for dynein in the present study was narrower than those for previous studies. One possible reason is much higher time resolution (100 μs) of our measurement than those of the previous studies. Improvement of time resolution would facilitate to detect fast stepping events. Other possibilities are that we used dynein from different species and/or used chimeric construct which has high affinity to the microtubule^29^. Off-axis step also showed narrower distribution than that of the previous reports^13,14^. In case of the off-axis step, in addition to the high time resolution, sub-nanometer localization precision would also facilitate to revolve the minimum step size of 5 nm clearly, which is 1.6 times smaller than that along on-axis (8 nm).

**Figure 3:**
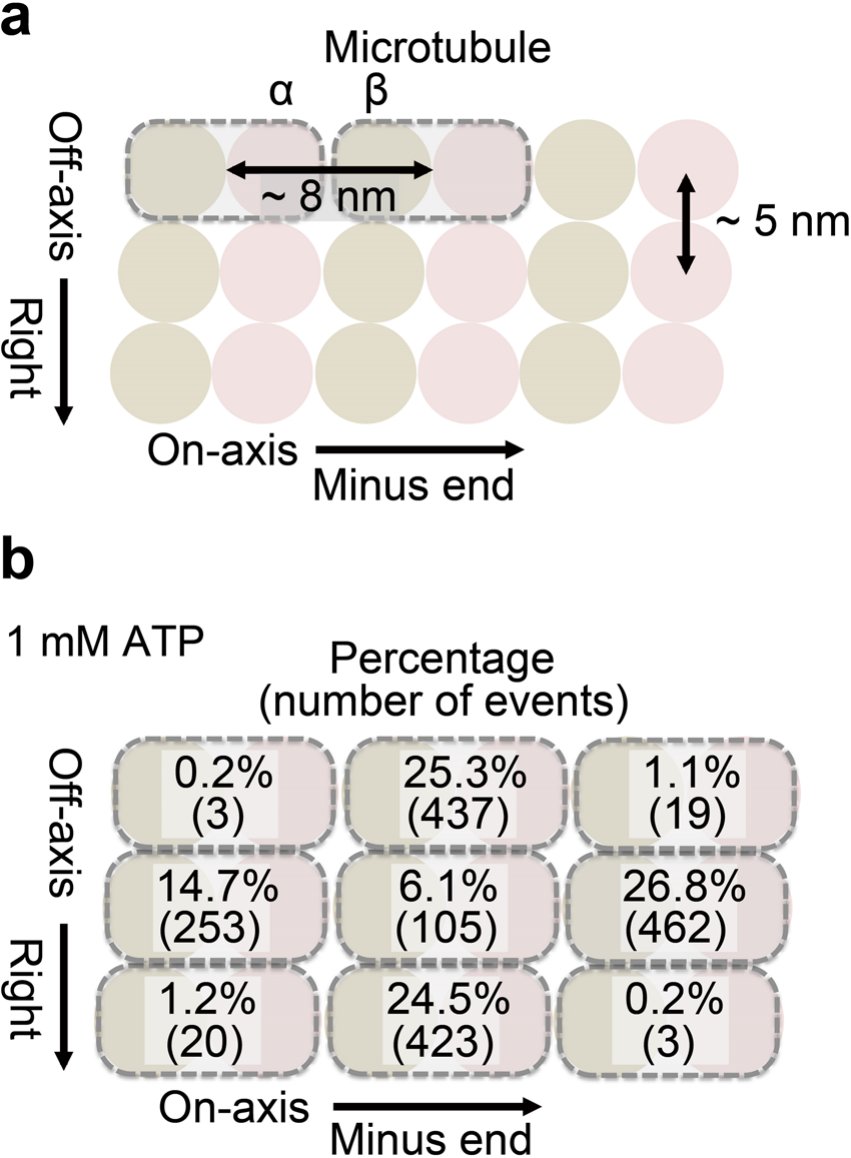
Preference of step direction in two dimensions at 1 mM ATP. (**a**) Schematic depiction of the binding cleft between αβ-tubulin dimer with pitches of 8 nm along on-axis (corresponding to the length of αβ-tubulin dimer) and 5 nm along off-axis (corresponding to the width of αβ-tubulin dimer)^33–35^. (**b**) Preference of the step direction at 1 mM ATP. Center portion represents an original binding site before step, and surrounding 8 positions represent next binding sites after step. Diagonal steps were also counted when the on- and off-axes steps occurred simultaneously. Small steps between −2 nm and +2 nm for both on- and off-axes were counted as rebinding to the original binding site. Note that fraction of diagonal step is slightly underestimated due to the limited precision of the step fitting.

Next we investigated preference of the step direction of the dynein in two dimensions. Figure 3a shows the schematic depiction of the binding cleft between αβ-tubulin dimers, with the pitches of 8 nm along on-axis (corresponding to the length of αβ-tubulin dimer) and 5 nm along off-axis (corresponding to the width of αβ-tubulin dimer)^33–35^. Figure 3b shows the preference of the step direction toward next binding sites at 1 mM ATP. Center portion represents an original binding site before step, and surrounding 8 positions represent next binding sites after step. In our analysis, diagonal steps were also counted when on- and off-axes steps occurred simultaneously, and small steps between −2 nm and +2 nm for both on- and off-axes were counted as rebinding to the original binding site. As results, on-axis step was twice biased to forward (26.8%) than backward (14.7%). Off-axis step was not biased to rightward (24.5%) or leftward (25.3%). Interestingly, fractions of forward, rightward and leftward steps were almost similar and only fraction of backward step was 1.7–1.8 times lower than other steps. Furthermore, diagonal steps were minor, where the total fraction was less than 3%. Rebinding to the original binding site was also minor (6.1%). These results indicate that only the backward steps are slightly suppressed compared with steps to other directions.

It is worth noting that in our analysis, we counted diagonal steps only when steps to on- and off-axes directions occur at the completely same frames with the time resolution of 100 μs. Considering the limited precision of the step fitting^32^, shift of few frames in fitting can occur and the fraction of the diagonal step seemed to be slightly underestimated. This point will be further discussed below, at the section of dwell time analysis.

### Dwell time analysis between steps

**Figure 4:**
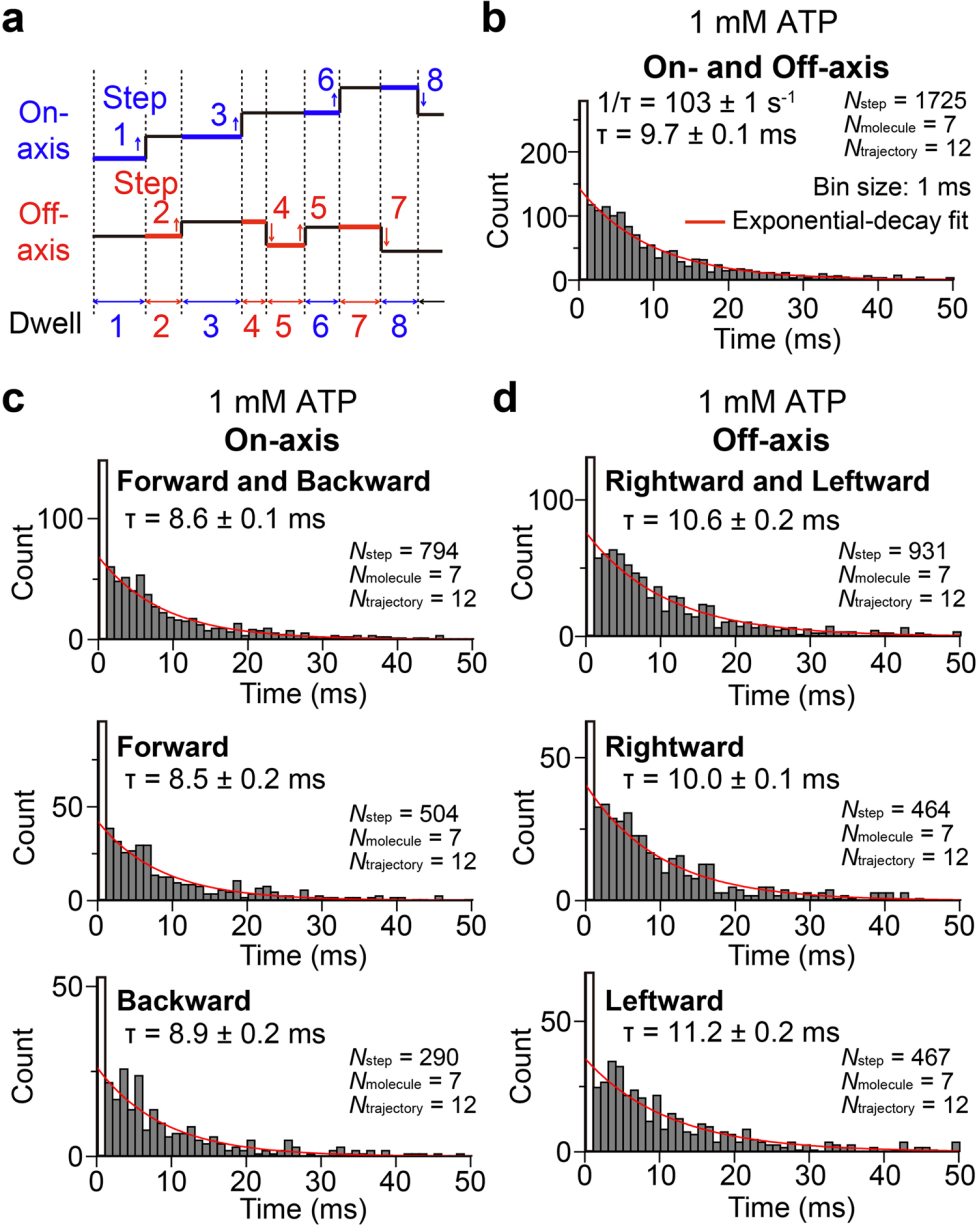
Dwell times between steps at 1 mM ATP. (**a**) Schematic depiction of the definition of dwell time. Steps along both on- and off-axes were mixed for the analysis. (**b**) Distribution of the dwell time along both on- and off-axes steps. The distribution was fitted with a single-exponential decay function (red line). For fitting, first bin was omitted. Rate constant was 103 s^−1^ (or 9.7 ms as time constant). (**c**) Distributions of the dwell time along on-axis steps before forward and backward (Top), before forward (middle), and before backward (bottom). Time constants were 8.6, 8.5, and 8.9 ms, respectively. (**d**) Distributions of the dwell time along off-axis steps before rightward and leftward (Top), before rightward (middle), and before leftward (bottom). Time constants were 10.6, 10.0, and 11.2 ms, respectively.

Figure 4 shows distribution of the dwell time between steps. For the dwell time analysis, both on- and off-axes steps were mixed (Fig. 4a), because we consider that each step along both on- and off-axes is coupled with same elementary steps of ATP hydrolysis reaction. As a result, we found that count of first bin (1 ms bin size) in the distribution was prominently high. If the first bin was omitted, the distribution was fitted well with a single-exponential decay function, consistent with uncoordinated stepping of each head in a dynein dimer as previously reported^13,14^. Rate constant obtained by the fitting was 103 ± 1 s^−1^ (fitted value ± fitting error, or 9.7 ± 0.1 ms as time constant, Fig. 4b). This value is close to the turnover number (*k*_cat_) of ATP hydrolysis for monomeric dynein (121 s^−1^)^36^, suggesting that each step is coupled with single ATP hydrolysis.

We attributed high count of the first bin in the dwell time distribution to the underestimation of the diagonal steps. If the diagonal steps are not detected correctly but counted as apparent individual steps to on- and off-axes due to the limited precision of the step fitting^32^, the dwell time between these steps will become very short and result in the high count at first bin. Fraction of the excess count at first bin (the excess count than the expected value estimated by the single-exponential decay fit) to the total number of steps (N_step_ = 1725) was 8.3% (N = 144), suggesting that diagonal steps are still minor event.

Next, we further classified the dwell times into four categories; dwell times before forward, backward, rightward, and leftward steps (Fig. 4c,d). The time constant along on-axis (forward and backward) showed only 1.2 times smaller value (8.6 ± 0.1 ms, Fig. 4c top) than that along off-axis (rightward and leftward) (10.6 ± 0.2 ms, Fig. 4d top). Furthermore, the time constant before forward steps was only 0.96 times smaller value (8.5 ± 0.2 ms) than that before backward steps (8.9 ± 0.2 ms), and the time constant before rightward steps was only 0.89 times smaller value (10.0 ± 0.1 ms) than that before leftward steps (11.2 ± 0.2 ms). These results indicate that the time constant before step does not largely change (less than 1.2 times difference) depending on their directions. Rate constants of the step for each direction were also calculated by the product of the rate constant for all directions (103 ± 1 s^−1^, Fig. 4b) and the fractions of the step for each direction. The obtained rate constants of forward and backward (on-axis) and rightward and leftward (off-axis) directions were 47 and 56 s^−1^, respectively, and those of forward, backward, rightward, and leftward directions were 30, 17, 28, and 28 s^−1^, respectively.

### [ATP] dependence of stepping behaviors

In addition to the physiologically-relevant high [ATP], 1 mM, we also observed motions of AuNP-labeled dynein at low [ATP] (100 and 10 μM) to investigate [ATP] dependence of the stepping behaviors (Figs. S2–S6). As [ATP] decreased, step size distributions along both on- and off-axes became slightly wider than that at 1 mM ATP, although elementary peaks around 8 nm and 5 nm were still major in on- and off-axes, respectively (Figs. S2 and S3). Slightly wider distribution of step size at low [ATP] may correlate with regulatory roles of AAA3 module^6^, because site occupancy will decrease as [ATP] decreases. Furthermore, preferences of the step direction in two dimensions at 100 and 10 μM ATP were basically similar to that at 1 mM ATP, indicating no [ATP] dependence of directional bias (Fig. S4). Analysis of the dwell time before steps also showed similar results to that at 1 mM ATP. The time constant did not largely depend on step directions, although the values of time constants increased as [ATP] decreased (Figs. S5 and S6). Then, also at low [ATP], rate constants of the step for each direction were calculated by the product of the rate constant for all directions (34 ± 1 and 23 ± 1 s^−1^ at 100 and 10 μM ATP, respectively, Figs. S5b and S6b) and the fractions of the step for each direction. The rate constants of forward and backward (on-axis), rightward and leftward (off-axis), forward, backward, rightward, and leftward directions were 17, 17, 12, 5, 8, and 9 s^−1^ at 100 μM ATP, and 11, 12, 7, 4, 6, and 6 s^−1^ at 10 μM ATP, respectively.

**Figure 5:**
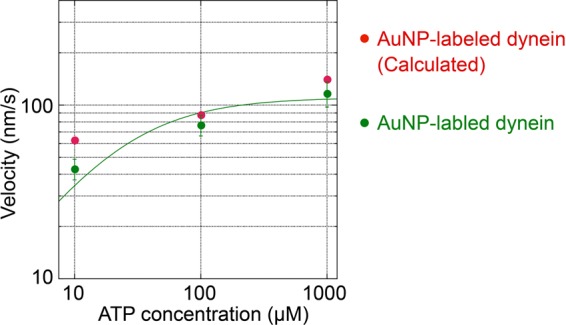
On-axis velocities of AuNP-labeled dynein as a function of [ATP]. Green circles indicate average velocities: 43 ± 6 nm/s, 77 ± 10 nm/s, and 117 ± 19 nm at 10 μM ATP (7 molecules, 9 trajectories), 100 μM ATP (8 molecules, 8 trajectories), and 1 mM ATP (7 molecules, 9 trajectories), respectively. The values of *K*_m_ and *V*_max_ obtained by the fitting with the Michaelis-Menten equation were 22 μM and 110 nm/s, respectively. Red circles are velocities calculated as products of on-axis average step sizes, and on-axis rate constants including both forward and backward steps: 63 nm/s, 88 nm/s, and 141 nm/s at 10 μM, 100 μM, and 1 mM ATP, respectively. Values used for calculations are: 5.7 nm, 5.2 nm, and 3.0 nm for on-axis average step sizes, and 11 s^−1^, 17 s^−1^, and 47 s^−1^ for on-axis rate constants, at 10 μM, 100 μM, and 1 mM ATP, respectively. Error bars represent standard errors.

We then compared velocity of motion in on-axis direction at different [ATP]s (Fig. 5). Average velocities were 43 ± 6 nm/s (mean ± standard error), 77 ± 10 nm/s, and 117 ± 19 nm/s at 10 μM, 100 μM, and 1 mM ATP, respectively (Fig. 5, green circles). The values of *K*_m_ and *V*_max_ obtained by fitting with the Michaelis−Menten equation were 22 μM and 110 nm/s, respectively. We also calculated velocities as products of on-axis average step sizes (Figs. 2c, S2b and S3b) and on-axis rate constants including both forward and backward steps. The values obtained were 63 nm/s, 88 nm/s, and 141 nm/s at 10 μM, 100 μM and 1 mM ATP, respectively (Fig. 5, red circles), and roughly agreed with the average velocity at each [ATP].

In our previous study of kinesin-1, unbound states of the head during stepping motion has been clearly observed with AuNP labeling^20^. On the other hand, in the present study of dynein, no clear unbound states (or one-head bound states) were observed at all [ATP] tried (Figs. 2a,b and S1–S3). This result is consistent with high duty ratio of dynein^29^. Another possible reason of no clear unbound states is the labeling position of the AuNP. In the present study, we labeled AAA2 of the head with AuNP, but this position is very far from the MTBD (>15 nm). This will make it more difficult to probe the unbound states. AuNP-labeling of the MTBD may visualize the unbound states more clearly.

### Possible stepping model and future perspective

**Figure 6:**
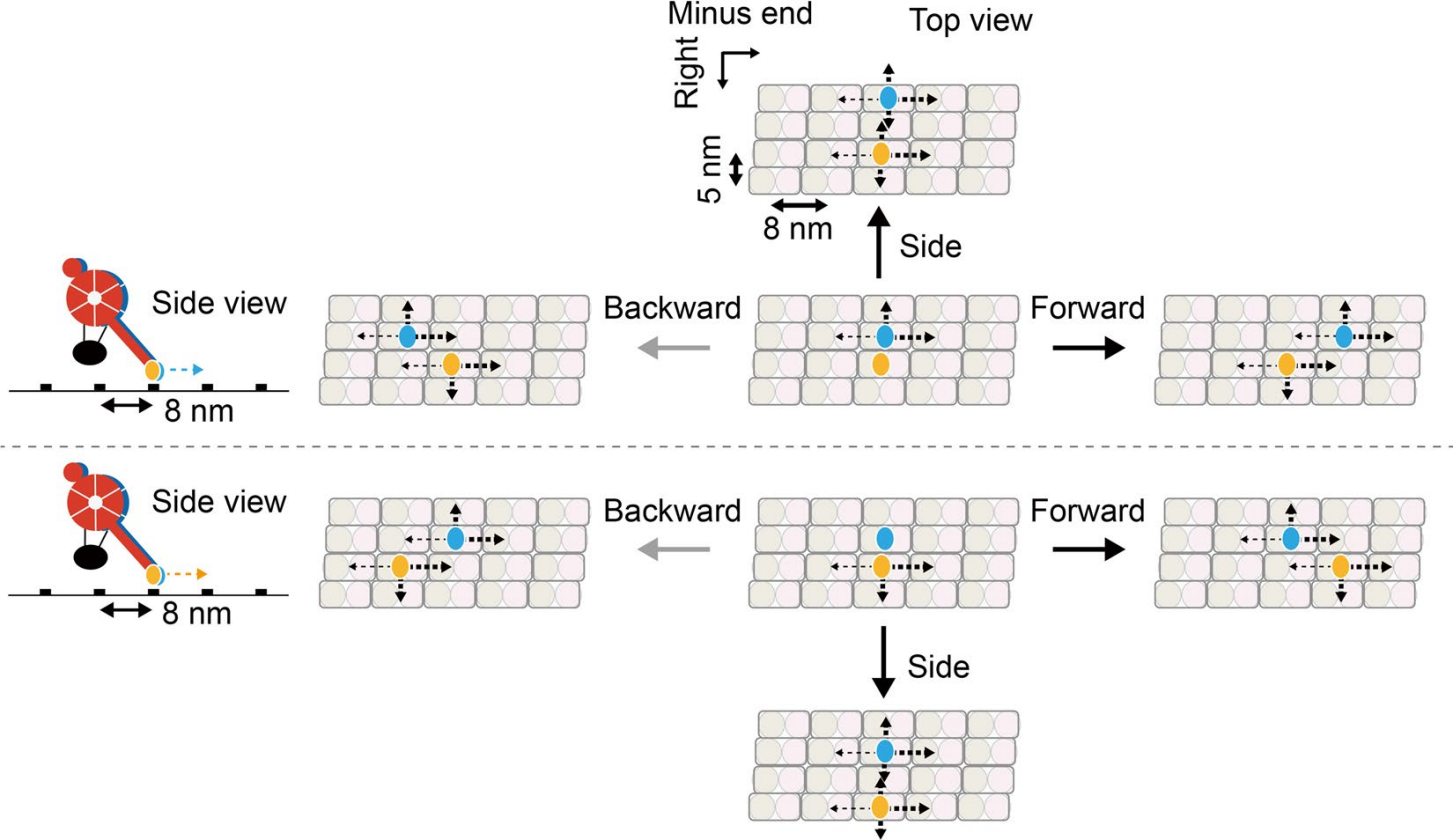
Schematic depictions of the possible stepping model of dynein. The head colored in red located at near side, that colored in blue located at far side. Each head moves to next binding sites through small stepping motion with the step sizes of ± 8 nm and ± 5 nm along on- and off-axes, respectively, matched with the minimum pitches of the binding cleft between αβ-tubulin dimers on the microtubule surface. Black arrows with dashed lines indicate possible moving directions of the head toward next binding sites. The state with two monomers bound on the same protofilament of the microtubule is excluded, because it has not been observed in the electron microscopic observations. In this model, only backward steps are slightly suppressed compared to forward and side steps. Moving directions of the head are partially restricted, depending on the location of another head.

Figure 6 shows schematic depictions of the possible stepping model of our chimeric dynein. In this model, the chimeric dynein exhibits small stepping motion in both on- and off-axes directions. This is because the step sizes matched well with the minimum pitches of the binding cleft of αβ-tubulin dimer on the microtubule (Fig. 2). During the motion, only the backward steps are slightly suppressed compared to other steps to forward and side directions (Fig. 3). In more detail, when the two heads are superimposed, direction of the off-axis step will be limited to rightward or leftward. As results, moving directions of the head will be partially restricted, depending on the location of another head. To our knowledge, dynein with two monomers bound on the same protofilament of the microtubule have not been observed in electron microscopic studies. Therefore, we excluded this configuration. Because distribution of the dwell time showed single-exponential decay (Fig. 4), we also expect that stepping motion of each head is uncoordinated as previously reported for yeast dynein^13,14^. If two heads are coordinated, the dwell time distribution will have a peak reflecting a consecutive reaction with two rate constants as the case for kinesin-1^15^. Furthermore, preference of the step direction (Figs. 3 and S4) and degree of the coordination (Figs. 4, S5 and S6) are almost independent on [ATP], and lifetime of the unbound states is always short.

It is worth noting that our model is consistent with the previous structural study of the same chimeric dynein construct bound to the microtubule^29^. In the previous structural analysis, about half of the dynein dimers showed closely superimposed heads and MTBDs, and the other half showed the heads separated each other and MTBDs both bound to the microtubule and roughly separated by 8 nm in on-axis direction. Furthermore, dynein molecules with one MTBD detached from the microtubule were not observed. The next important question is whether our stepping model can be applied to the wild-type processive dynein not only from *D. discoideum* but also from yeast, requiring experimental verifications with our imaging method.

In this study, we have successfully observed stepping motion of single head of the chimeric dynein dimer with microsecond time resolution and sub-nanometer localization precision. However, as shown in previous studies^13,14^, to completely understand coordination (or uncoordination) between two heads in a dynein dimer, high-speed simultaneous observation of both heads will be essential. For this purpose, we have recently developed multicolor high-speed imaging system for silver, gold, and silver–gold alloy nanoparticle probes^37^. To successfully apply our new method, further decrease of the probe size will be necessary to minimize the steric hinderance between two nanoparticles attached to each head of the dynein, while keeping the time resolution and localization precision. In the future, simultaneous dual-color imaging of both heads of dynein with microsecond and sub-nanometer resolution will be required to better understand the mechanism of dynein stepping.

## Methods

### Expression, purification and biotinylation of chimeric dynein construct

The artificially-dimerized chimeric dynein construct (Fig. 1a) was expressed in cells derived from the *D. discoideum* Ax2 strain. The experimental procedures for the protein expression and purification were same with those of the previous study^29^. The head of dynein was biotinylated via SNAP-tag introduced into the AAA2 module, by incubating 2.6 μM dynein (concentration as monomer) with 33 μM SNAP-biotin (S9110S, New England BioLabs) at a molar ratio of 13 (SNAP-biotin/dynein molecules) for 1 h at 30 °C. Unreacted SNAP-biotin was removed by micro-bio-spin P-30 gel column (7326223, Bio-Rad) with centrifugation at 1,000 × *g* for 4 min at 4 °C. Dynein solution was drop-frozen and stored in liquid nitrogen. Frozen drops were quickly thawed before experiments.

### Estimation of biotinylation ratio with dot blot

Labeling ratio of biotin on dynein was estimated by dot blot. On a dried PVDF membrane (WSE-4051, ATTO), 5 μL of methanol was deposited, and subsequently, 10 μL of 100 nM biotinylated dynein (concentration as dimer) was deposited. As a reference, that of 25, 50 and 75 nM biotinylated kinesin-1-S55C (concentration as dimer)^20^ was also deposited on the same membrane at different locations. After drying, the membrane was immersed in methanol for 1 min, washed with PBS buffer with 0.1% Tween20, and immersed in blocking buffer (EzBlock Chemi, ATTO) for 17.5 h at 4 °C. After blocking, the membrane was immersed into a solution of an anti-biotin HRP-linked antibody (#7075, Cell Signaling Technology) diluted with blocking buffer at a ratio of 1:2000, and incubated for 1 h. The membrane was washed with Tris-buffered saline with 0.1% Tween20, and stained with 3,3′,5,5′-tetramethylbenzidine (TMB) (AE-1490 EzWestBlue, ATTO) solution for 30 s. The membrane was then washed with water, and dried for observation. Image of the membrane was taken by a digital camera, and analyzed by ImageJ software. When there was a biotinylated protein on the membrane, the spot appeared darker than the surrounding area of the membrane due to TMB staining. To quantify the amount of protein on the spots, image contrast was inverted to have a positive contrast at where the biotinylated protein exists. Signal intensity of the spot was obtained from the image area including spot. Background intensity obtained from the neighboring area without spot was subtracted from signal intensity of the spot. Signal intensities of the spots of biotinylated kinesin-1-S55C increased with protein concentration, and linear fit was used as a calibration curve. From signal intensity of dynein spot and the calibration curve, biotinylation ratio of dynein was estimated as 0.4 per head, under the assumption that biotinylation ratio of kinesin-1-S55C was 1.0 per head.

### Surface modification of AuNP with streptavidin

AuNP with nominal diameter of 30 nm was purchased from British Biocell International Solutions (EMGC30, BBI). The actual diameter was 30.8 ± 2.4 nm, as previously observed with an electron microscope^31^. The procedure for surface modification of 30 nm AuNP with streptavidin was same as previously reported method with small modifications^31^. In short, the incubation time with alkanethiol mixture solution was changed to 5 h. The time for centrifugation to remove unreacted alkanethiols and streptavidin was changed to 5 min at 10,000 × *g*.

### Annular illumination total internal reflection dark-field microscopy with axicon lens

A continuous-wave 532 nm laser (Excel, Laser Quantum, UK) was used for an illumination light source of dark-field imaging. We constructed annular illumination total internal reflection dark-field microscopy with perforated mirror and axicon lens, as reported previously^31^. The ring-shaped beam was formed by introducing laser beam into axicon lens (α = 10 °). The ring-shaped laser beam was introduced into the side port of the inverted microscope (IX70, Olympus), and reflected by the peripheral region of the perforated mirror. The size of the hole along short and long axes was 7.0 and 9.9 mm, respectively. The ring-shaped laser beam was focused at the back-focal plane of the objective lens (NA 1.49, APON 60XOTIRF, Olympus) to form total internal reflection at the interface of glass and water. The sample on the glass surface was illuminated by the evanescent field near the interface. Scattered light from the AuNP near the glass substrate was collected by the same objective lens, passed through the hole of the perforated mirror, and detected by high-speed CMOS camera (Fastcam AX100, Photron). The image was 5 × or 6.7 × magnified, and image pixel size was 67.6 nm/pixel or 50.3 nm/pixel, respectively. Sequential dark-field images were obtained to analyze the motion of AuNP-labeled dynein at time resolution of 100 μs (10,000 frame per second). The laser intensity was measured before objective lens, and that at the sample plane was calculated as the power density (μW/μm^2^) with the assumption of 100% transmittance of the objective lens. For observation, the laser intensity at the sample plane was set at 40 μW/μm^2^. Under this condition, 30 nm AuNP fixed on the glass surface showed localization precision of 0.7 nm at 100 μs time resolution.

### Preparation of microtubules

To obtain microtubules for single-molecule observation, we purified tubulin from pig brain at 6 mg/mL^38^. The aliquots of the purified tubulin was stored in −80 °C, and was used for polymerization before experiment. To prepare microtubule, the tubulin was polymerized at 37 °C in the presence of 0.75 mM GTP, and the solution was centrifuged at 72,000 rpm for 30 min at 37 °C. The supernatant was removed, and the pellet was dissolved in BRB80 buffer (80 mM PIPES, 1 mM MgCl_2_, 1 mM EGTA, pH 6.8) with 1 mM GTP and 20 μM taxol.

### Single-molecule imaging of dynein motion on microtubule

The flow cell for single-molecule imaging was constructed by two coverslips (upper side: 18 mm × 18 mm, lower side: 24 mm × 32 mm, Matsunami Glass). They were attached by grease with thin spacers to form three flow cells in parallel. The surface of the lower side coverslip was modified with (3-aminopropyl)triethoxysilane (LS-3150, Shin-Etsu Silicone) before flow cell construction, using same method described previously^31^. Then, constructed flow cell was incubated with 8% glutaraldehyde (072–0262, Wako) for 20 min, and washed well with water. Subsequently, the microtubule suspension was introduced and incubated for 2 min. After fixation of the microtubules on the glass surface of the flow cell, remaining microtubules were washed with BRB80 buffer with 20 μM taxol. To suppress non-specific binding of dynein, BRB80 buffer containing 20 μM taxol and 1 mg/ml bovine serum albumin (BSA, 010-15153, Wako) was introduced into the flow cell and incubated for 2 min. Excess amount of BSA, remaining in solution, was removed by the observation buffer which consists of P30 buffer (30 mM PIPES, 2 mM MgCl_2_, 1 mM EGTA, pH 7.0), 0.4 mg/mL casein, 2 mM tris(2-carboxyethyl)phosphine) (TCEP), 1% pluronic F-127, 1 mM MgSO_4_, and ATP (1 mM, 100 μM, or 10 μM). As an ATP regenerating system, 1 mM creatine phosphate and 0.2 mg/ml creatine kinase or 2.5 mM phosphoenolpyruvic acid and 0.1 mg/ml pyruvate kinase were also added in the observation buffer. Then, streptavidin-coated 30 nm AuNP and biotinylated dynein were mixed in a tube at a molar ratio of 3 (AuNPs/dynein dimer), incubated for 10 min, and introduced into the microtubule-fixed flow cell for observation. Final concentration of dynein was set at 150 pM, and that of ATP was 1 mM, 100 μM, or 10 μM. All incubations and observations were carried out at room temperature (25 ± 1 °C).

### Analysis of dynein motion

At each frame of the sequential dark-field image, the center coordinate of the AuNP-labeled dynein was obtained by two-dimensional Gaussian fit to the scattering image of AuNP. The X and Y axes were adjusted to the microtubule short- and long-axes by the method same as previous report^20^. For the analysis of step, the trajectories along both on- and off-axes were treated with a median filter (window size of 20 frames) to reduce the noise. The SD of the median-filtered trajectory along both on- and off-axes was calculated for each time frame with the window size of ± 20 frames. The steps along both on- and off-axes were identified by the algorithm developed by Kerssemakers *et al*.^32^. From the identified steps, step sizes were measured independently for on- and off-axes directions. Preference of the step direction was calculated by counting the number of the steps to the directions of surrounding 8 positions from the original binding site. Small steps between –2 nm and +2 nm for both on- and off-axes were counted as rebinding to the original binding site. Diagonal steps were counted when both on- and off-axes steps occurred simultaneously at the same time frame. For the dwell time analysis, both on- and off-axes steps were mixed to obtain dwell time between all steps. The dwell time was further classified into four categories; dwell times before forward, backward, rightward, and leftward steps. The distributions of the dwell time were fitted with a single-exponential decay function to obtain time and rate constants. Velocity of the motion of dynein along on-axis was obtained by the linear fit of on-axis trajectory with time.

## Supplementary information

Supplementary information.

## Data Availability

The datasets generated during and/or analyzed during the current study are available from the corresponding author on reasonable request.
