# Supplementary material for: Small stepping motion of processive dynein revealed by load-free high-speed single-particle tracking
Source: Sci Rep. 2020 Jan 23;10:1080. doi: 10.1038/s41598-020-58070-y (PMC6978368; doi:10.1038/s41598-020-58070-y)
Supplement: Supplementary file 1 — Supplementary information. [file 41598_2020_58070_MOESM1_ESM.pdf]

Supplementary Information

Small stepping motion of processive dynein revealed by load-free high-speed single-particle tracking

Jun Ando[1,2], Tomohiro Shima[3], Riko Kanazawa[4], Rieko Shimo-Kon[4], Akihiko Nakamura[1,2], Mayuko Yamamoto[1], Takahide Kon[4], and Ryota Iino[1,2,]*

[1]Institute for Molecular Science, National Institutes of Natural Sciences, Okazaki 444-8787, Aichi, Japan

[2]The Graduate University for Advanced Studies (SOKENDAI), Hayama 240-0193, Kanagawa, Japan

[3]Department of Biological Sciences, Graduate School of Science, The University of Tokyo, Hongo 113-0033, Tokyo, Japan

[4]Department of Biological Sciences, Graduate School of Science, Osaka University, Toyonaka, Osaka, 560-0043, Japan.

*Correspondence: iino@ims.ac.jp

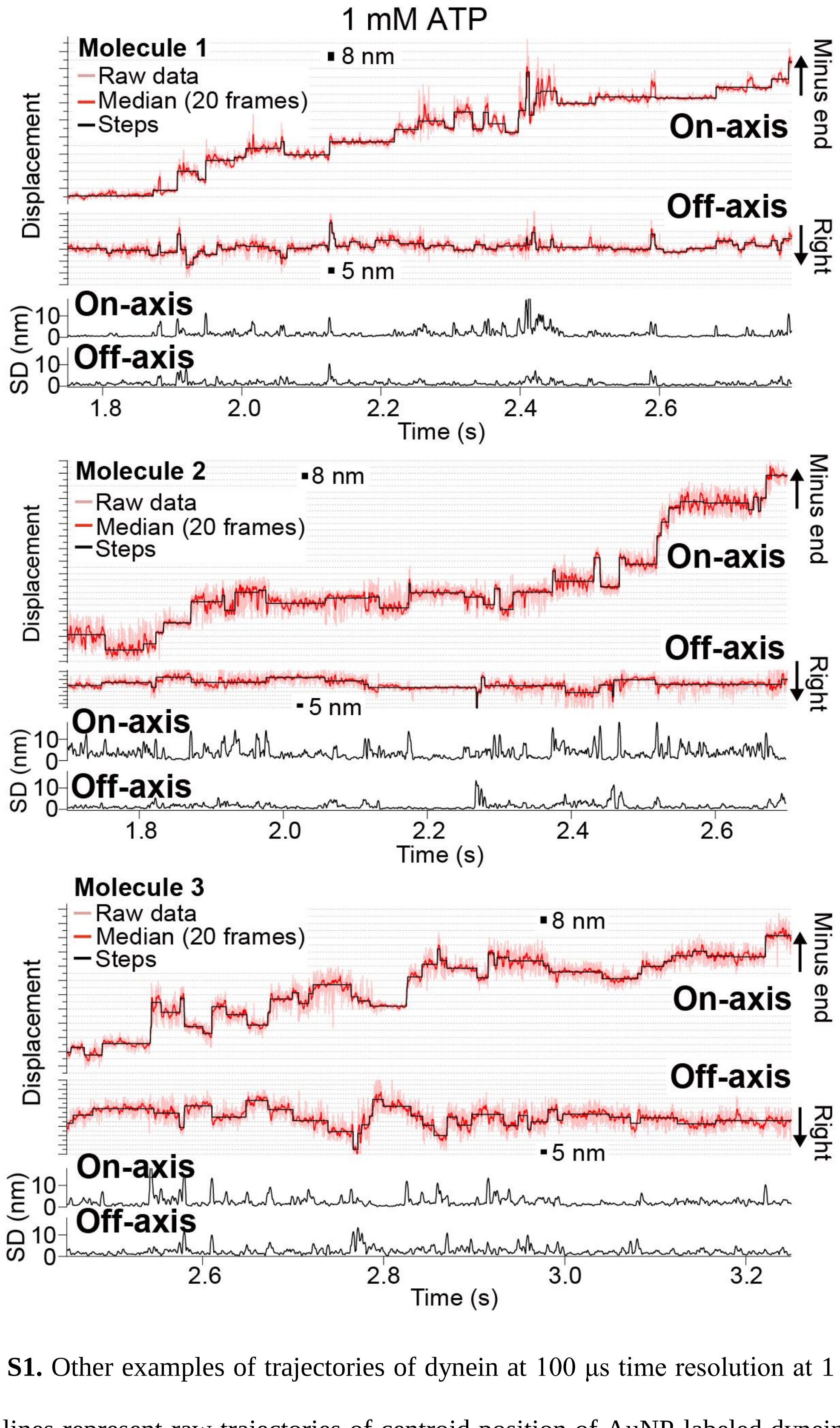

FIGURE S1. Other examples of trajectories of dynein at 100 μs time resolution at 1 mM ATP. Light red lines represent raw trajectories of centroid position of AuNP-labeled dynein along the

microtubule long axis (on-axis) and short axis (off-axis). Red lines represent median-filtered trajectories (window size of 20 frames). Lower panel shows SD of the median-filtered trajectory along the on- and off-axes at each time frame t, calculated for t ± 20 frames. Black lines show steps and pauses in the median-filtered trajectories identified by the algorithm developed by Kerssemakers *et al.*[1].

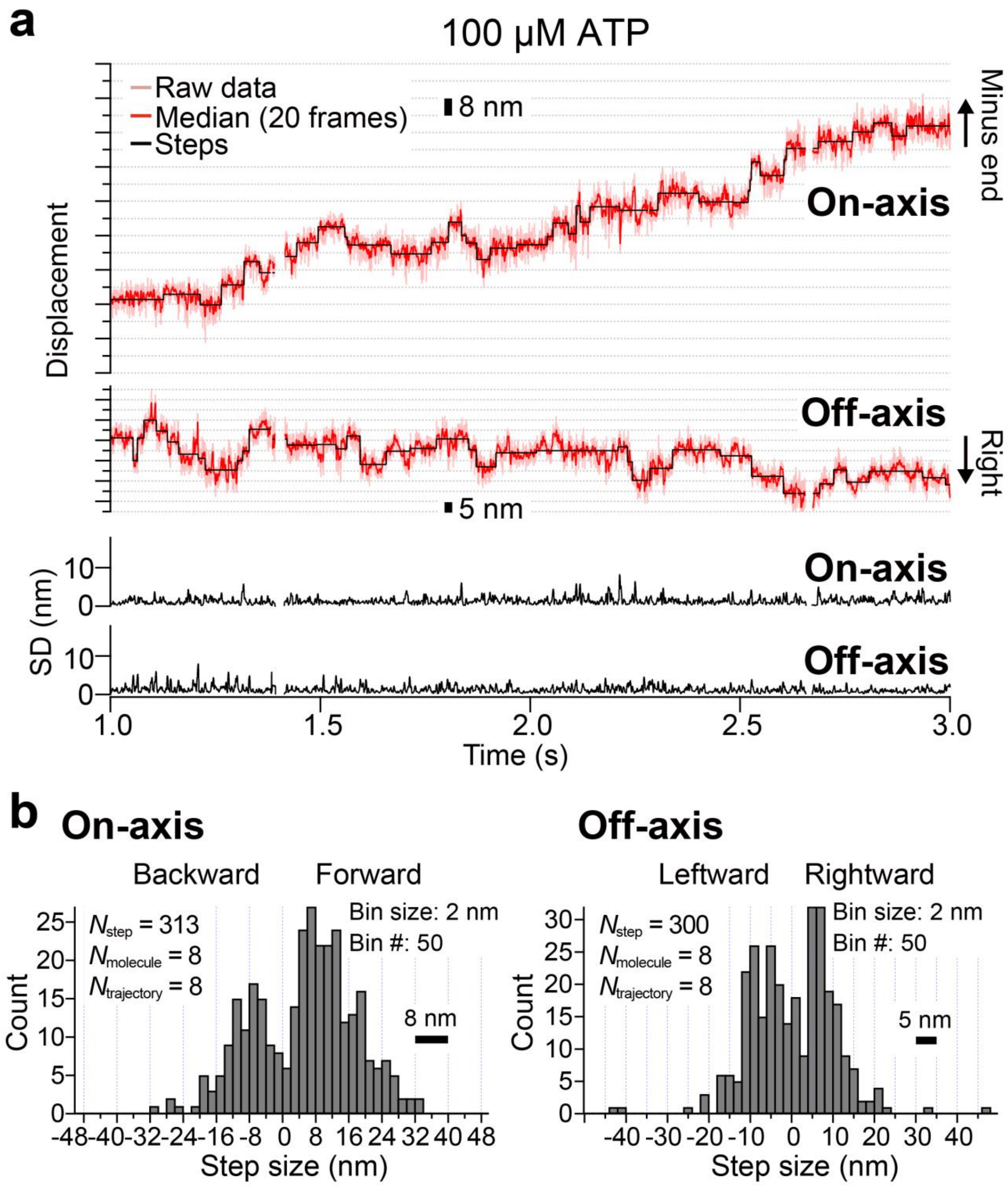

FIGURE S2. Trajectory and step size at 100 μs time resolution at 100 μM ATP. (a) Light red lines represent typical raw trajectories of centroid position of AuNP-labeled dynein along the microtubule long axis (on-axis) and short axis (off-axis). Red lines represent median-filtered

trajectories (window size of 20 frames). Lower panel shows SD of the median-filtered trajectory along the on- and off-axes at each time frame t, calculated for t ± 20 frames. Black lines show steps and pauses in the median-filtered trajectories identified by the algorithm developed by Kerssemakers *et al.*[1]. (b) Distributions of step size along on- and off-axes.

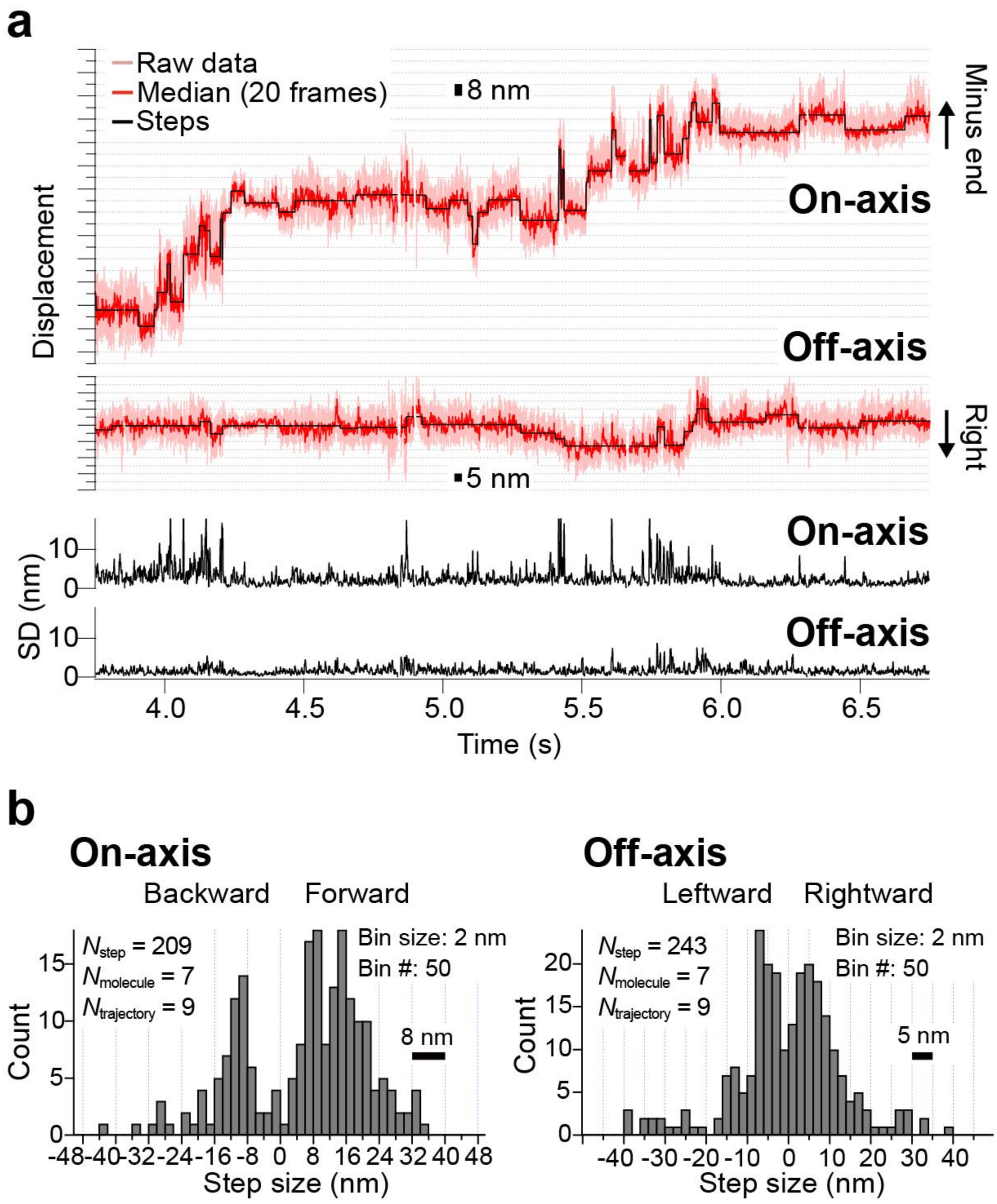

FIGURE S3. Trajectory and step size at 100 μs time resolution at 10 μM ATP. (a) Light red lines represent typical raw trajectories of centroid position of AuNP-labeled dynein along the microtubule long axis (on-axis) and short axis (off-axis). Red lines represent median-filtered

trajectories (window size of 20 frames). Lower panel shows SD of the median-filtered trajectory along the on- and off-axes at each time frame t, calculated for t ± 20 frames. Black lines show steps and pauses in the median-filtered trajectories identified by the algorithm developed by Kerssemakers *et al.*[1]. (b) Distributions of step size along on- and off-axes.

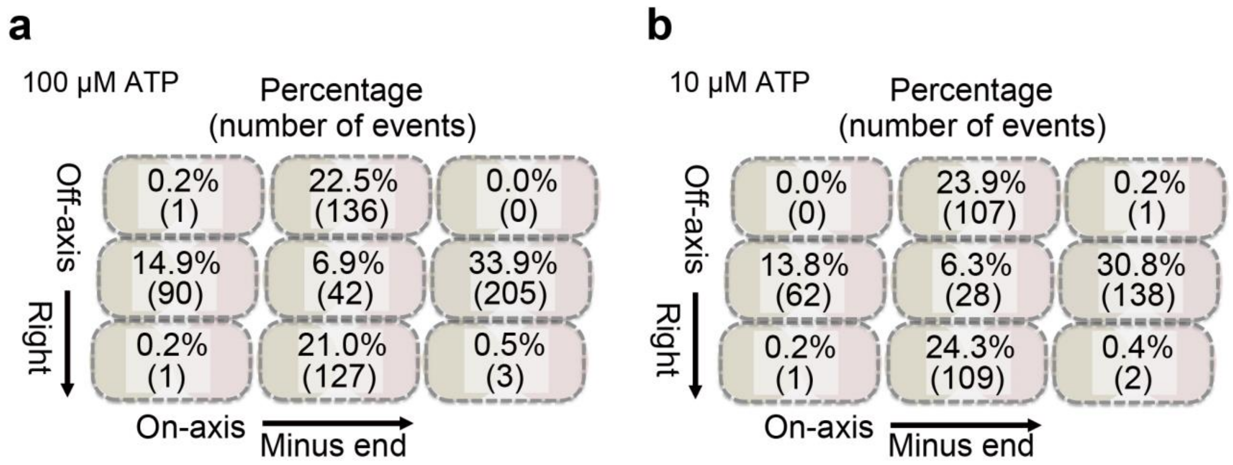

FIGURE S4. Preference of step direction in two dimensions at 100 μM (a) and 10 μM (b) ATP. Center portion represent an original binding site before step, and surrounding 8 positions represent next binding sites after step. Diagonal steps were also counted when the on- and off-axes steps occurred simultaneously. Small steps between –2 nm and +2 nm for both on- and off-axes were counted as rebinding to the original binding site. Note that each position along on-axis includes not only 8 nm steps but also 16 nm or larger steps, and each position along off-axis includes not only 5 nm steps but also 10 nm or larger steps. Also note that fraction of diagonal step is slightly underestimated due to the limited precision of the step fitting.

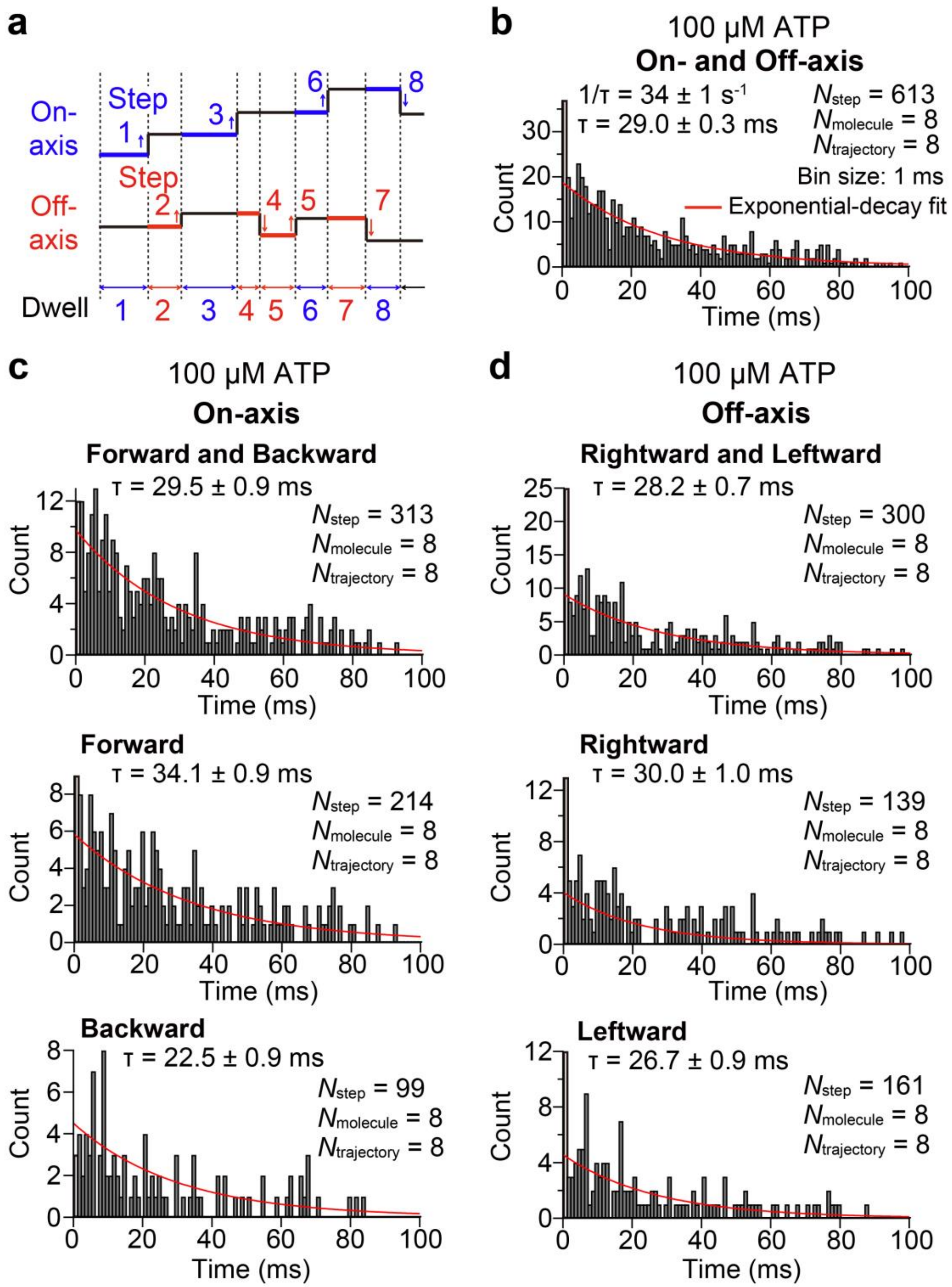

FIGURE S5. Dwell times between steps at 100 μM ATP. (a) Schematic depiction of the definition of dwell time. Steps along both on- and off-axes were mixed for the analysis. (b) Distribution of the dwell time along both on- and off-axes steps. The distribution was fitted with

a single-exponential decay function (red line). For fitting, first bin was omitted. Rate constant was 34 s^{-1} (or 29.0 ms as time constant). (c) Distributions of the dwell time along on-axis steps before forward and backward (Top), before forward (middle), and before backward (bottom). Time constants were 29.5, 34.1, and 22.5 ms, respectively. (d) Distributions of the dwell time along off-axis steps before rightward and leftward (Top), before rightward (middle), and before leftward (bottom). Time constants were 28.2, 30.0, and 26.7 ms, respectively.

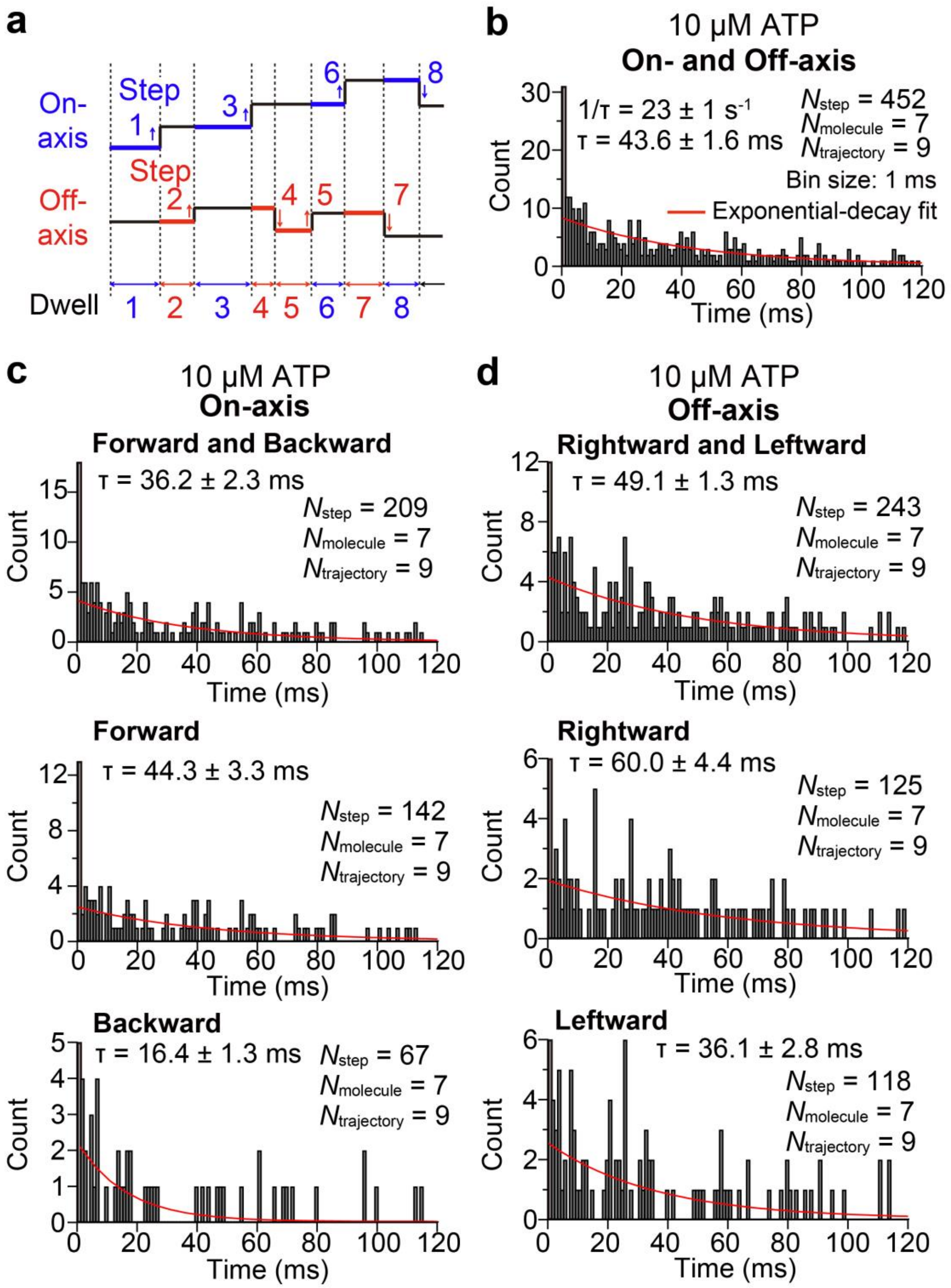

FIGURE S6. Dwell times between steps at 10 μM ATP. (a) Schematic depiction of the definition of dwell time. Steps along both on- and off-axes were mixed for the analysis. (b) Distribution of the dwell time along both on- and off-axes steps. The distribution was fitted with

a single-exponential decay function (red line). For fitting, first bin was omitted. Rate constant was 23 s^{-1} (or 43.6 ms as time constant). (c) Distributions of the dwell time along on-axis steps before forward and backward (Top), before forward (middle), and before backward (bottom). Time constants were 36.2, 44.3, and 16.4 ms, respectively. (d) Distributions of the dwell time along off-axis steps before rightward and leftward (Top), before rightward (middle), and before leftward (bottom). Time constants were 49.1, 60.0, and 36.1 ms, respectively.

Supplementary References

1. Kerssemakers, J. W. J. *et al.* Assembly dynamics of microtubules at molecular resolution. *Nature* **442**, 709-712, doi:10.1038/nature04928 (2006).
